# Explaining the effects of an intervention designed to promote evidence-based diabetes care: a theory-based process evaluation of a pragmatic cluster randomised controlled trial

**DOI:** 10.1186/1748-5908-3-50

**Published:** 2008-11-19

**Authors:** Jillian J Francis, Martin P Eccles, Marie Johnston, Paula Whitty, Jeremy M Grimshaw, Eileen FS Kaner, Liz Smith, Anne Walker

**Affiliations:** 1 Health Services Research Unit, University of Aberdeen, Aberdeen, UK; 2 Institute of Health and Society, Newcastle University, Newcastle upon Tyne, UK; 3 College of Life Sciences and Medicine, University of Aberdeen, Aberdeen, UK; 4 Clinical Epidemiology Program, Ottawa Health Research Institute and Department of Medicine, University of Ottawa, Ontario, Canada; 5 Faculty of Medical Sciences, Newcastle University, Newcastle upon Tyne, UK; 6 Manchester Business School, University of Manchester, Manchester, UK

## Abstract

**Background:**

The results of randomised controlled trials can be usefully illuminated by studies of the processes by which they achieve their effects. The Theory of Planned Behaviour (TPB) offers a framework for conducting such studies. This study used TPB to explore the observed effects in a pragmatic cluster randomised controlled trial of a structured recall and prompting intervention to increase evidence-based diabetes care that was conducted in three Primary Care Trusts in England.

**Methods:**

All general practitioners and nurses in practices involved in the trial were sent a postal questionnaire at the end of the intervention period, based on the TPB (predictor variables: attitude; subjective norm; perceived behavioural control, or PBC). It focussed on three clinical behaviours recommended in diabetes care: measuring blood pressure; inspecting feet; and prescribing statins. Multivariate analyses of variance and multiple regression analyses were used to explore changes in cognitions and thereby better understand trial effects.

**Results:**

Fifty-nine general medical practitioners and 53 practice nurses (intervention: n = 55, 41.98% of trial participants; control: n = 57, 38.26% of trial participants) completed the questionnaire. There were no differences between groups in mean scores for attitudes, subjective norms, PBC or intentions. Control group clinicians had 'normatively-driven' intentions (*i.e*., related to subjective norm scores), whereas intervention group clinicians had 'attitudinally-driven' intentions (*i.e*., related to attitude scores) for foot inspection and statin prescription. After controlling for effects of the three predictor variables, this group difference was significant for foot inspection behaviour (trial group × attitude interaction, beta = 0.72, p < 0.05; trial group × subjective norm interaction, beta = -0.65, p < 0.05).

**Conclusion:**

Attitudinally-driven intentions are proposed to be more consistently translated into action than normatively-driven intentions. This proposition was supported by the findings, thus offering an interpretation of the trial effects. This analytic approach demonstrates the potential of the TPB to explain trial effects in terms of different relationships between variables rather than differences in mean scores. This study illustrates the use of theory-based process evaluation to uncover processes underlying change in implementation trials.

## Background

There is broad, international agreement over what constitutes high quality health care for people with diabetes [1,2]. In the UK, this has been enshrined in a National Service Framework for people with diabetes [3]. However, the most efficient method of delivering care remains unclear [4]. A recent systematic review [4] of quality improvement interventions to improve the quality of care in patients with diabetes showed that a range of different interventions resulted in small to modest improvements in glycaemic control and in provider adherence to optimal care. However it also identified important methodological concerns, including evidence of publication bias.

Given the variety of possibly effective interventions, it may be instructive to focus on possible mechanisms underlying intervention effects, with the goal of identifying how such interventions may work. This type of process evaluation can lead to the identification of general principles that will help to optimise interventions. The study reported here was a theory-based process evaluation of a pragmatic cluster randomised controlled trial design. The trial evaluated the effectiveness and efficiency of an area wide 'extended' computerised diabetes register incorporating a full structured recall and management system, actively involving patients and including individualised patient management prompts to primary care clinicians based on locally-adapted evidence-based guidelines.

Three Primary Care Trusts (PCTs) (geographically based organisational units that are directly responsible for health care) served by a district hospital-based diabetes register had produced improvements in the quality of care, but performance had later plateaued leaving scope for further improvement. The opportunity arose to extend the computerised diabetes register to a full structured recall and management system.

The development and implementation of the Diabetes Recall and Management System (DREAM) intervention has been described in detail elsewhere [5,6]. In summary, the pre-existing diabetes register functioned as a central register of patients with diabetes. A structured dataset was completed on paper forms and returned to the central register; from these data, both patient-specific and aggregated information were provided annually to patients and clinicians. This system was enhanced in five ways. The software was enhanced by incorporating locally adapted national evidence-based guidelines. The functionality of the system was enhanced to provide: automated prompts to patients and primary care clinicians that a review consultation was necessary; a structured management sheet (including patient-specific management suggestions); an enhanced monitoring system to follow up reasons for non-attendance from both patients and clinicians and to re-schedule appointments, based on non-return of a completed management sheet; and patient feedback for patients in primary care. Because of difficulties operating this element of the software, it was not possible to run the final feature during the lifetime of the trial.

Alongside this trial, a process evaluation study was conducted. In the literature about randomised controlled trials, process evaluation may focus on one or more of three groups of issues:

1. Quality control, fidelity, or coverage (*i.e*., was the intervention successfully and consistently implemented?).

2. Acceptability of the intervention from the participants' perspective.

3. Explanatory modelling: an exploration of processes underlying change (or lack of change) following a successfully implemented intervention.

**Figure 1:**
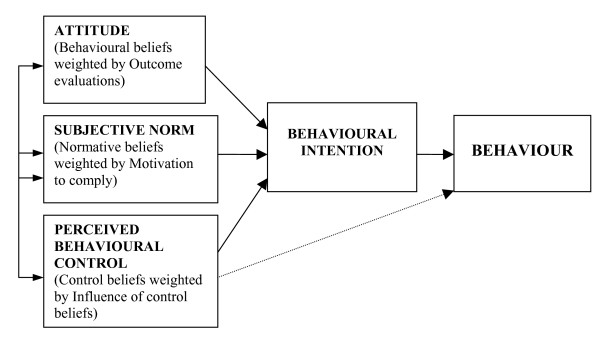
**The theory of planned behaviour (Ajzen, 1991)**. Attitude = being in favour of, or against, doing something (the behaviour). Subjective norm = perceived pressure to do, or not to do, the behaviour. Perceived Behavioural Control = perception of having, or not having, control over the behaviour.

The study reported here investigated the third of these: processes underlying change or lack of change to assess possible reasons for the success or lack of success of the intervention. The evaluation was based on the Theory of Planned Behaviour (TPB) [7]. The TPB proposes a model about how human action is guided. It predicts the occurrence of a specific behaviour, provided that the behaviour is intentional (*i.e*., the model does not claim to predict behaviours that are habitual or automatic). The model is increasingly being used to predict intentions and behaviour with respect to clinical actions [8]. The TPB model is depicted in Figure 1 and represents the three cognitive variables that the theory suggests will predict the intention, which is the precursor of behaviour. Because this process evaluation was conducted at the end of the intervention period, we do not claim that the cognitive variables caused a change in behaviour. We distinguish between prediction –, something that researchers do when they know one score (*e.g*., attitude) and want to estimate another (*e.g*., intention) – and causation (*i.e*., when one factor is brought about by another, independently of whether the factors are measured). By using a model which is predictive in this sense, we may illuminate processes underlying the trial results.

The TPB is predicated on careful specification of the behaviour under investigation. The behaviour is defined in terms of its target, action, context and time (TACT). For example, for the clinical behaviour of measuring a patient's blood pressure, the target is the patient; the action is taking the blood pressure reading; the context is the clinical consultation; and the time may be expressed in terms of frequency (*e.g*., every time the patient visits the surgery; at least once every six months) or delay (*e.g*., at the patient's next routine visit to the surgery; within the next six months). In the current study, three behaviours were identified from the clinical guidelines used in the DREAM Trial as being central in the management of Type 2 diabetes: measuring blood pressure, inspecting feet, and prescribing statins (to lower cholesterol). As data about actual behaviour were not available at the level of the individual clinician (but only at the level of primary care practices), we used the measure of intention as the dependent variable for this process evaluation. A recent systematic review concluded that intention is an appropriate proxy measure of individual behaviour [9].

The findings of the trial are reported elsewhere [10], but the findings relevant to these three behaviours were: first, patients in intervention practices were significantly more likely than patients in control practices to have a recording of having had either a foot check or a measurement of blood pressure but not a measurement of serum cholesterol; and second, the mean cholesterol level in patients from intervention practices was significantly lower than in control practices, but there was no difference between intervention and control groups in the levels of blood pressure recorded.

The aim of this study was to elucidate the cognitions of health professionals that underlay these selected clinical behaviours. We did this in two ways: first, by testing for differences in cognitions between the intervention and control groups of the trial and second, by identifying the patterns of association within each trial group and comparing these with effects of the intervention on clinical practice [10].

## Methods

### Development of the questionnaire

The three 'predictor' variables in the TPB are attitudes (being in favour of, or against doing something); subjective norms (perceived pressure from social sources to do, or not to do something); perceived behavioural control, or PBC (perception of having, or not having control over the behaviour). They may be measured 'directly' by asking responders to summarise their overall attitude, perceived pressure and so on, or 'indirectly', by asking responders about specific beliefs and combining the answers in a manner specified by the theory [7]. According to the TPB, when using direct measures in a regression analysis to predict intention, adding the indirect measures should not increase the level of prediction. However, we included both measures in the questionnaire to test this part of the theory, because direct and indirect measurement approaches make different assumptions about the underlying cognitive structures [11]. Briefly, indirect measures are based on responses to items about specific beliefs and scores are then combined by the researcher. The assumptions are that the method used for combining responses (weighting and then averaging the scores) reflects the methods that individuals use when forming, for example, an attitude, and that all relevant beliefs have been represented among the questionnaire items. Direct measurement effectively asks individuals themselves to combine the separate beliefs. It does not rely on the assumption that all relevant beliefs have been represented in the questionnaire but assumes that people can accurately combine and report a global attitude, subjective norm, and perceived level of control over the behaviour in question. The construction of the measures of the three predictor variables and of intention was based on standard practice in the field including the advice of researchers [7,11-13].

To construct the indirect measures we first conducted a qualitative study. A member of the research team (LS) interviewed 12 general practitioners (GPs) and practice nurses not involved in the DREAM trial about the behaviours under investigation. We designed the schedule for these semi-structured interviews to elicit responders' beliefs relating to the constructs of the TPB. Both GPs and practice nurses were encouraged to talk freely about these beliefs, and any ambiguities were clarified using appropriate prompts. Interviews were tape recorded and transcribed. Answers to questions were entered into response tables. We identified the most frequently mentioned beliefs and used them to develop items for indirect measurement of the three predictor variables.

We developed a questionnaire for each of the three behaviours. The response format for all items was a seven-point Likert-type scale, from 1 (strongly agree) to 7 (strongly disagree). We pre-tested this initial draft of the questionnaire with six GPs not involved in the DREAM trial for style and clarity of content and to determine completion time. Minor revisions of wording were made to the questionnaire in the light of their comments. Responses were explored for range, and items with low variance were eliminated from the final questionnaire, because they would be unlikely to discriminate within the analysis. The final questionnaire consisted of 154 items, including questions about the size of practices and demographic details. Sample questions are presented in Table 1; the full questionnaire is available as Additional file 1.

**Table 1:** Sample questionnaire items for the constructs relating to measuring blood pressure.

Construct	Sample item
Attitude (direct)	Overall I think measuring these patients' blood pressure is beneficial to them

Attitude (indirect)	(If I measure a patient's blood pressure, I will detect any problems at an early stage) × (Detecting any problems at an early stage is very important)

Subjective norm (direct)	People who are important to me think that I should measure the blood pressure of my patients with diabetes

Subjective norm (indirect)	(Patients would approve of me measuring their blood pressure) × (Patients' approval of my practice is of importance to me)

Perceived behavioural control (direct)	Measuring patients' blood pressure is easy

Perceived behavioural control (indirect)	(If the patient has high blood pressure they think they have another illness as well as diabetes) × (If the patients did not see raised blood pressure as a separate illness to diabetes I would be more likely to measure their blood pressure)

Intention	I intend to measure the blood pressure of most of the patients' with diabetes that I see during the next month

### Procedure

The questionnaire was mailed to all 280 GPs and practice nurses in the DREAM trial. Two reminder letters were sent to non-responders at fortnightly intervals.

### Psychometric properties of the questionnaire

Internal consistency coefficients were calculated for the intention measure and for the direct measure of attitude, for each of the three behaviours. Coefficient alpha was satisfactory (between 0.87 and 0.98). Direct measures of subjective norm and perceived behaviour control were two-item measures, and so consistency was assessed using Pearson's correlation coefficients. Using a criterion for acceptability of *r *> 0.25, internal consistency was high (*r *> 0.4) for the measures of subjective norms and mixed (two coefficients close to zero) for the measures of PBC. It is not appropriate to use an internal consistency criterion for assessing the reliability of indirect measures, as the objective in using these measures is to sample a diverse range of beliefs [11].

Because the two PBC items did not have adequate internal consistency, it was not valid to combine these scores for the main analysis. We selected one item ('Overall, I feel that I can [do X]') to represent PBC for the analysis as it was more consistently related to the other TPB variables across the three behaviours.

Indirect measures were computed using the 'multiplicative composite' approach suggested by Ajzen and Fishbein [13]. That is, the score for each belief was multiplied by the score of its perceived importance weight (see Figure 1) and the resulting products were summed to give a total score for attitude, subjective norms, and PBC for each of the three behaviours. For direct and indirect measures, scores were scaled so that a low score always indicated a more positive attitude, intention, etc.

There is considerable debate in the TPB literature about whether to use response scales of 1 to 7 or -3 to +3 in the multiplicative composite approach (*e.g*., French and Hankins [14]). As the questionnaire was lengthy, we decided, on a pragmatic basis, to use a consistent 1–7 response format to minimise responder fatigue.

## Results

### Non-response Analysis

**Figure 2:**
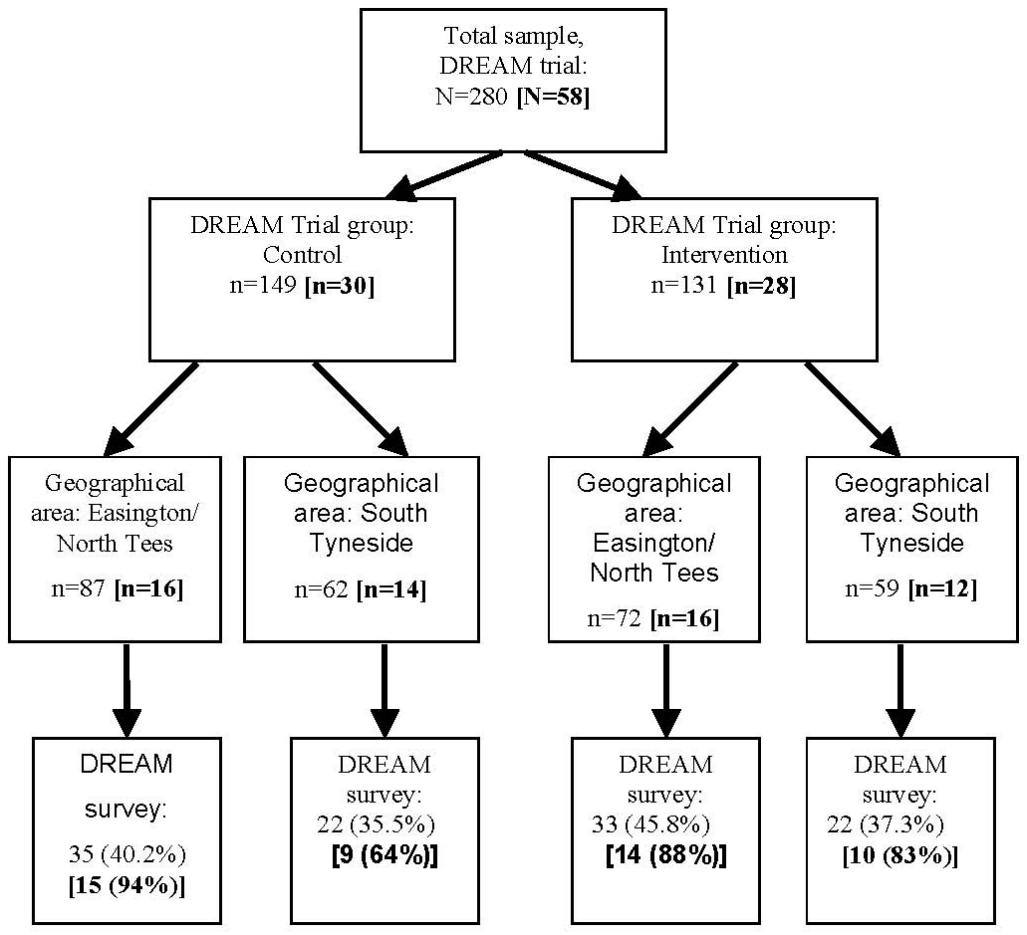
Individual-level and [practice-level] response rates, DREAM survey.

Figure 2 presents the response rates for the survey relative to the trial, for the intervention and control groups. Overall, the individual response rate was 40.0% (intervention: 42.6%; control: 37.7%). The practice level response rate (at least one responder in the practice) was 81.0% (intervention: 82.1%; control: 80.0%). We used a series of chi-square analyses to compare responders and non-responders on variables that could be accessed for non-responders. These showed no associations with trial group, register, gender, professional role, or working in a training practice (all *p *> 0.05). However, responders had been qualified for significantly longer than non-responders (M = 22.46 years and M = 18.92 years, respectively; 95% confidence interval for mean difference: 0.42 – 6.66).

Chi-square analyses of responders showed no association between trial group (intervention versus control) and the following variables: diabetes register, number of GPs in the practice, number of nurses in the practice, prescribing status of nurses, and years since qualified (all *p *> 0.3). Nevertheless, as this was a process evaluation of a trial, the remaining descriptive analyses were conducted separately for intervention and control participants.

### Initial analyses

Bivariate correlations between the direct measures for each of the three behaviours are presented, separately for the intervention and control groups, in Table 2. Means, standard deviations, and correlations between the direct and indirect measures of the same construct are also included. These correlations may be used to assess the content validity of the indirect measures. If the indirect measures were individually relevant and together adequately represented the range of beliefs, this should result in moderate-to-strong positive correlations between direct and indirect measures. Using this criterion, validity of the indirect measures was acceptable for attitudes and subjective norms, but questionable for PBC. That is, it is possible that, to create a questionnaire of acceptable length, we may have excluded important control beliefs that influence clinicians' perceptions of control over these behaviours.

**Table 2:** Means, standard deviations (SD), and correlations between the predictor variables (direct measures) and intention scores and indirect measures, for each of the three behaviours, computed separately for the intervention and control groups.

	Control Group: Direct Measures			Intervention group: Direct Measures		
	
	Attitude	Subj Norm	PBC	Attitude	Subj Norm	PBC
**Measuring Blood Pressure**						

Subjective Norm	0.52**	-		0.25	-	
PBC	0.56**	0.36**	-	0.15	0.25	-
Intention	0.47**	0.21	0.47**	0.28*	0.07	0.39**

Indirect Measure	0.57**	0.43*	0.29*	0.34*	0.39*	-0.07

Mean (sd)	1.62(0.67)	2.62(1.33)	2.69(1.81)	1.41(0.63)	2.22(1.32)	2.70(1.97)

**Foot examination**						

Subjective Norm	0.63**	-		0.49**	-	
PBC	0.26	0.22	-	0.12	0.25	-
Intention	0.61**	0.77**	0.33*	0.28*	0.48**	0.14

Indirect Measure	0.70**	0.66*	-0.03	0.64*	0.54*	0.13

Mean (sd)	2.12(0.91)	2.99(1.44)	3.00(1.51)	1.96(1.09)	2.64(1.43)	3.04(1.73)

**Prescribing statins**						

Subjective Norm	0.52**	-		0.61**	-	
PBC	0.50**	0.52**	-	0.44**	0.56**	-
Intention	0.31*	0.48**	0.44**	0.42*	0.36**	0.41**

Indirect Measure	0.37**	0.52*	0.32*	0.28*	0.36*	0.07

Mean (sd)	2.44(1.12)	2.94(1.27)	2.83(1.54)	2.19(1.12)	2.66(1.39)	2.62(1.52)

*p < 0.05, **p < 0.01.

PBC = Perceived Behavioural Control; Subj Norm = Subjective Norm.

Lower mean scores reflect stronger positive attitudes towards the behaviour, stronger perceived social pressure to enact the behaviour and greater perceived control over the behaviour.

### Group differences in TPB variables: Multiple analyses of variance (MANOVAs)

To identify factors affecting the mean values of the TPB variables, a series of MANOVAs were conducted. For each of the three behaviours under investigation, intention and direct measure scores for the three predictor variables were entered as dependent variables. Three designs were used:

Trial group (intervention; control) × job title (GP; nurse) × PCT)

Trial group (intervention; control) × practice size (< 4 GPs; ≥ 4 GPs)

Trial group (intervention; control) × years since qualified (≤ 23 years; > 23 years)

There was no main effect of trial group and no interaction effects involving trial group on the profile of TPB scores. That is, the intervention appears to have had no effect on scores for attitudes, subjective norms, PBC, or intentions. However, there was a main effect of practice size on intentions. Responders (both GPs and nurses) in smaller practices had stronger intentions to measure blood pressure. In addition, there was a main effect of job title (GP; nurse) on cognitions. Nurses had more positive intentions and attitudes than GPs for measuring BP and examining feet. The pattern for statins was reversed, with GPs reporting stronger intentions, more positive attitudes, and also greater PBC than nurses. This again lends support to the criterion validity of the PBC item, as it would be expected that nurses, most of whom are not eligible to prescribe, would report lower control over prescribing behaviour. No other main effects or interaction effects were significant in the MANOVAs.

### Predicting intention: Regression analyses

A multiple linear regression analysis on intentions for each of the three behaviours was carried out separately for the intervention and control groups (Table 3). At the first step, the direct measures of Attitude, Subjective Norm, and PBC were entered; indirect measures were entered at the second step. This was to check whether the solution would change depending on which method of measurement (direct or indirect) was used. In the intervention group, the addition of indirect measures did not improve prediction of intention for any of the three behaviours. In the control group, the addition of indirect measures did not improve prediction of intention to prescribe statins or to examine feet. However, prediction of intention to measure blood pressure did improve when indirect measures were added (R^2 ^change = 0.14, *p *< 0.05). The significant predictor at the second step was attitude (indirect), β = 0.47, *p *= .002. Although this finding relates to only one of six regression analyses performed, it suggests the possibility that clinicians in the control group and intervention group may have been thinking about their beliefs and intentions in different ways.

**Table 3:** Results of regression analyses on intentions for three behaviours, with direct measures entered at Step 1 and indirect measures entered at Step 2, for control and intervention groups.

Dependent variable	Independent variables	Step 1			Step 2	
Control Group						

		β	R	Adj R^2^	R^2 ^change	R^2^

Intention to measure blood pressure	Attitude	0.28				
	Subjective	-0.09				
	Norm	0.39*				
	PBC		0.56***	0.28	0.14*	0.42
						
Intention to inspect feet	Attitude	0.17				
	Subjective	0.67***				
	Norm	0.16				
	PBC		0.80***	0.64	0.03	0.67
						
Intention to prescribe statins	Attitude	0.04				
	Subjective	0.41*				
	Norm	0.13				
	PBC		0.50**	0.20	0.06	0.26

Intervention Group						

Intention to measure blood pressure	Attitude	0.28*				
	Subjective	-0.13				
	Norm	0.50***				
	PBC		0.56***	0.27	0.01	0.28
						
Intention to inspect feet	Attitude	0.54***				
	Subjective	0.21				
	Norm	0.02				
	PBC		0.67***	0.42	0.03	0.45
						
Intention to prescribe statins	Attitude	0.33*				
	Subjective	0.06				
	Norm	0.40**				
	PBC		0.62***	0.34	0.03	0.37

*p < 0.05, **p < 0.01, ***p < 0.001

PBC = Perceived behavioural control.

We performed a second series of hierarchical regression analyses, with professional (GP or nurse) and practice size (< 4 GPs; ≥ 4 GPs) entered at the first step and the direct TPB measures entered at the second step. After controlling for job title and practice size, the TPB predictor variables again significantly added to the variance in intention explained, and the patterns of significant predictors were similar to the first set of analyses. This finding was consistent in both the control and intervention group for all three behaviours.

### Interpreting trial effects

Table 3 shows a consistent pattern that may represent an effect of the intervention. Specifically, for inspecting feet and prescribing statins there was a trend for intentions to be predicted most strongly by subjective norms in the control group and by attitudes in the intervention group. To determine whether these trends were reliable, two further hierarchical regression analyses were performed in which the interactions between trial group and attitude, and between trial group and subjective norm, were entered in the second step (Table 4). In the analysis relating to the inspection of feet, intention was predicted not only by the main effect of subjective norm (β = 0.97) but also by both interactions. The directions of the interaction effects indicate that subjective norm was a stronger predictor of intention in the control group than in the intervention group. Conversely, attitude was a stronger predictor of intention in the intervention group than in the control group. Neither interaction term was significant in the analysis predicting intention to prescribe statins.

**Table 4:** Results of regression analyses (n = 112) on intentions to inspect feet and intentions to prescribe statins, with direct measures entered at Step 1 and the interaction between direct measures and trial group entered at Step 2.

Dependent variable	Independent variables	Step 1			Step 2		
		β	R	Adj R^2^	β	R^2 ^change	R^2^

Intention to inspect feet	Attitude	0.42***			-0.21		
	Subjective Norm (SN)	0.38***			0.97***		
	PBC	0.06			0.08		
			0.71***	0.48			
	Trial group × Attitude				0.72*		
	Trial group × SN				-0.65*	0.03*	0.51

Intention to prescribe statins	Attitude	0.09					
	Subjective Norm	0.67**					
	PBC	0.16					
			0.52***	0.25		0.02	0.27

*p < 0.05, **p < 0.01, ***p < 0.001. PBC = Perceived behavioural control. Control group coded '0; intervention group coded '1'.

## Discussion

This study was a theory-based process evaluation undertaken to investigate the cognitive processes underlying trial effects. In addition, extraneous variables that may affect clinicians' cognitions and behaviour were investigated, and patterns of clinical behaviours were explored. Results are discussed below in four sections: trial effects; effects of demographic factors on cognitions about clinical behaviours; limitations of the study; and general conclusions.

### Trial effects

In the context of the effect of interventions on cognitive variables, there are two possible types of effect. First, an intervention may alter the mean values of scores on predictor variables. Second, an intervention may alter the relationships between the cognitive predictor variables and an outcome, in this case, intentions. It appears that the effect of the intervention in the DREAM trial was of the second kind. With respect to inspecting feet, subjective norms were more strongly related to intention in the control group than in the intervention group, and attitudes were more strongly related to intention in the intervention group than in the control group. Thus, at the end of the intervention, intentions to inspect feet were 'normatively-driven' in the control group but 'attitudinally-driven' in the intervention group.

There are a number of possible explanations for how the intervention might have strengthened the attitude-intention relationship. It could be that clinicians became more familiar with the content of the guideline by receiving the intervention, and that this familiarity (or additional knowledge) strengthened the consistency between attitudes and intentions. In addition, when attitudes are based on direct experience, they are more likely to be brought to mind in the relevant context [15]. Furthermore, it may be that the systematic application of the guideline in terms of prompts relating to specific patients made the importance of key clinical indicators more salient. For all of these possibilities, attitudes would supersede subjective norms as the primary predictor of intentions.

There is empirical evidence that attitudinally-driven intentions are more likely to be translated into action than normatively-driven intentions [16-18]. This has been explained by reference to self-determination theory [19], which distinguishes between self-determined (or intrinsically motivated) and externally controlled regulation of intention. Within this framework, attitudes represent internal pressure (one's own views), whereas subjective norms represent external pressure (the perceived views of others), to act. Self-determination theory proposes that internalised motivation is related to the stability of intentions and is a better predictor of behaviour than motivation arising from external sources [20,21].

The implication of this in the context of the DREAM trial is as follows. On the basis of these findings, we would expect higher levels of foot inspection to be recorded in the intervention group than in the control group. This is what was found in the trial. Thus, the results of the current study support the principle that attitudinally-driven intentions are better translated into action than are normatively-driven intentions. A further key trial effect (lowered cholesterol among participants in the intervention group) was possibly related to the trend (albeit non-significant in our analysis) for statin prescription also to be attitudinally-driven (*i.e*., internally motivated) in the intervention group.

A strength of this study was that we investigated three behaviours among the same population at the same time. This enabled us to compare and contrast patterns of prediction across the behaviours. The three selected behaviours were strongly contrasting: the measurement of blood pressure is a frequently enacted behaviour (intention data in this study showed restricted range and possible ceiling effects); the inspection of feet is a clinical action that both physicians and their practice nurses are qualified to do (although our data showed that cognitions of the two professional groups tend to be different); and the prescription of statins is restricted to physicians and a small proportion of nurses. Yet the TPB was effective in predicting intentions to do these contrasting behaviours, suggesting that the model was stable across a range of behaviours

### Effects of demographic factors

Demographic factors appeared to affect cognitions about clinical behaviours. As may be expected from the different responsibilities assigned to different roles, professional role (GP versus nurse) influenced cognitions. Nurses had stronger positive attitudes and intentions to measure blood pressure and inspect feet, whereas for prescribing statins, GPs had stronger attitudes, perceived control, and intentions. This would be expected and supports the validity of the measurement instruments.

A further demographic effect was practice size. In smaller practices, GPs and nurses had stronger intentions to measure blood pressure, and GPs had stronger intentions to prescribe statins. This is consistent with the results of a previous trial that tested the effects of educational outreach in a primary care setting [22], and suggests that a fruitful approach to exploring the implementation of evidence-based practice could include an investigation of organisational factors in relation to practice size.

## Limitations

This study had a number of limitations. First, although the non-response analysis indicated that the sample was representative in all but one of the measured variables (number of years since professional qualification), the response rate was low (40% at the individual level; 81% at the practice level). It is also possible that some responders were completing the questionnaire on behalf of other practice staff and this would introduce measurement error. Second, the items measuring PBC had poor psychometric properties, and so their reliability is uncertain. Third, TPB constructs were measured only once – after the intervention. However, randomisation of primary care practices to the intervention and control groups in the trial should have resulted in similar cognitions between groups before the intervention took place. Finally, as the process evaluation study sampled only three clinical behaviours out of all the appropriate behaviours relating to this complex intervention, there may have been other mechanisms underlying trial effects that were not detected by this study. For example, it may have been that the automated prompts to patients and clinicians regarding the need for a review consultation resulted in increased concordance in interactions between patients and clinicians and that this increased patients' involvement in the management of their condition.

## Conclusion

This theory-based process evaluation of the DREAM trial explored cognitions about three clinical behaviours relating to the management of diabetes to identify possible effects of the trial intervention on cognitions, including intentions. Independently of whether participants were in the intervention group or control group, professional role and practice size influenced attitudes, PBC, and intentions. This suggests that interventions that are directed to entire practices or health care teams may have different effects on individuals within those teams and across different organisational structures. Finally, it appears that the intervention strengthened the link between attitudes and intentions towards inspecting feet, with similar non-significant trends in cognitions about prescribing statins. This stronger link between internal pressures (attitudes) and intentions than between external pressures (subjective norms) and intentions was associated with trial outcomes. This lends support to the principle that when intentions are driven by attitudes rather than perceived social pressure, those intentions are more likely to be translated into action. Thus, understanding the difference between attitudes and subjective norms allowed us to understand some of the intervention effects. This study thus illustrates the use of theory-based process evaluation to uncover processes underlying change in implementation trials.

## Competing interests

The authors declare that they have no competing interests.

## Authors' contributions

MPE, AW, JMG and MJ developed the idea for the study. MPE, AW, LS, MJ and PW developed the instruments and conducted the data collection. JJF, MPE, MJ, conducted the analyses. JJF, MPE, MJ, PW, JMG and EK contributed to the interpretation of the analyses and the drafting of the paper. All authors have seen and approved the final draft.

## Supplementary Material

Additional file 1 **DREAM PE Final Questionnaire.** This questionnaire is the theory-based instrument that was used to collect the data for the DREAM process evaluation study. Click here for file
